# Variation in rotavirus vaccine coverage by sub-counties in Kenya

**DOI:** 10.1186/s41182-017-0051-z

**Published:** 2017-04-24

**Authors:** Ernest Apondi Wandera, Shah Mohammad, John Odhiambo Ouko, James Yatitch, Koki Taniguchi, Yoshio Ichinose

**Affiliations:** 1 KEMRI/Nagasaki University, Institute of Tropical Medicine, Kenya Research Station, P.O. Box 19993-00202, Nairobi, Kenya; 2 Leading Program, Graduate School of Biomedical Sciences, Nagasaki University, Nagasaki, Japan; 3 Public Health Department, Kiambu sub-county, Kiambu, Kenya; 4 Department of Virology and Parasitology, School of Medicine, Fujita Health University, Toyoake, Japan

**Keywords:** Kenya, Rotavirus vaccine, Administrative data, Coverage, Dropout

## Abstract

Rotavirus gastroenteritis is an important cause of childhood morbidity and mortality in Kenya. In July 2014, Kenya introduced the rotavirus vaccine into her national immunization program. Although immunization coverage is crucial in assessing the real-world impact of this vaccine, variability in the vaccine coverage across the country is likely to occur. In view of this, we estimated the extent of coverage for the rotavirus vaccine at two socio-economically different sub-counties using the administrative data. The findings indicate disparities in vaccine coverage and access between the sub-counties and, thus, underscore the need to strengthen immunization systems to facilitate timely, accessible, and equitable vaccine delivery across the country. Both sub-counties recorded high vaccine dropout, suggestive of poor utilization of the vaccine. In this regard, increased social mobilization is needed to encourage vaccine compliance and to enhance tracking of vaccine defaulters. While efforts to improve the accuracy of the administrative coverage estimates are crucial, vaccination coverage surveys will be needed to verify the administrative coverage data and help identify specific factors relating to rotavirus vaccine coverage in the country.

## Background

The rotavirus is the most common cause of severe diarrhea among children <5 years globally and is estimated to cause 215,000 deaths annually [1]. In Kenya, the rotavirus is estimated to cause more than 3908 deaths [1], 3015 outpatient visits, and 279 hospitalizations per 100,000 children <5 years [2] and to cost the healthcare system US$ 10.8 million annually [3]. Safe and effective rotavirus vaccines are considered to be important tools for reducing the burden of diarrhea and, thereby, contributing to the achievement of the Sustainable Development Goal 3 [4, 5]. In July 2014, Kenya introduced Rotarix®, a two-dose rotavirus vaccine into her Expanded Program on Immunization (EPI). The vaccine is administered orally at 6 and 10 weeks of age [6]. Over the first 20 years of its introduction in Kenya, the rotavirus vaccine is predicted to avert 60,935 undiscounted deaths and 216,454 hospital admissions among children aged <5 years and help the government avoid a healthcare cost of US$ 30 million and a cost per disability-adjusted life year (DALY) of US$ 38 million [7].

Nevertheless, a high vaccination coverage is essential to maximize the impact of the rotavirus vaccine. In countries with high levels of immunization coverage, the impact of rotavirus vaccines in reducing the burden of severe childhood diarrhea has been remarkable [8–10]. Conversely, in low-income settings like Kenya, vaccine coverage rates are likely to be lower due to programmatic, geographical, and social challenges [11]. According to the WHO/UNICEF [12], the rotavirus vaccine achieved only 38% coverage in 50% of the national target Kenyan population in 2014 and 66% in 2015. There was a countrywide dropout rate of 5.8% with eight counties reporting >10% dropout. However, given the fact that Kenya is a socio-economically diverse country [13], variability in rotavirus vaccine coverage across the sub-counties would not be surprising. In view of this, we estimated rotavirus vaccine coverage at Kiambu, a peri-urban sub-county, and Mbita, a rural sub-county.

## Main text

### Methods

Kiambu sub-county is located in Kiambu County, Central Kenya, on the outskirts of Nairobi, the capital of Kenya. According to the World Bank ranking, Kiambu is the second richest county in Kenya, with an estimated GDP of 3.0 billion US dollars (USD), which accounts for 11% of the national GDP [13]. The major economic activities in the sub-county are agriculture and industries. According to the 2014 Kenya Demographic and Health Survey (KDHS) [14], 82.8% of children aged 12–23 months in this area were fully vaccinated. Mbita sub-county is located on the shores of Lake Victoria in Homa Bay County, Western Kenya, and about 400 km west of Nairobi. Homa Bay County is among the poorest in Kenya with its GDP of 0.3 billion USD accounting for only 1.2% of the national GDP [13]. Less than 25% of the adult population has a regular wage employment. The population depends on subsistence farming, small-scale businesses, fishing, and keeping domestic animals [15]. According to the 2014 KDHS, only 53.7% of children aged 12–23 months in this area were fully immunized [14].

We obtained the administrative data on rotavirus vaccination from the Units of Vaccines and Immunization Services (UVISs) for Kiambu and Mbita sub-counties. The administrative data included the target population aged <1 year which is eligible for rotavirus vaccination, the monthly targets for rotavirus vaccination, and the monthly doses (1 and 2) of rotavirus vaccine routinely administered to the target population in each of the sub-counties as summarized in Table 1. Using these data, we estimated the percentage of rotavirus immunization coverage and dropout rates in Kiambu and Mbita sub-counties between August 2014 and April 2016. Coverage, which is the proportion of vaccinated individuals among the target population, was calculated by dividing the number of individuals vaccinated with rotavirus vaccine dose 1 or 2 (numerator) by the number of individuals targeted for vaccination (denominator) within the same period (month/year). This proportion was then multiplied by 100 to obtain the percentage coverage. Dropout rate, which refers to the number of individuals who start an immunization schedule but fail to get the last dose on the schedule, was calculated by subtracting the number of individuals who received the last dose of rotavirus vaccine from the number of individuals who received the first dose of the vaccine (numerator) and dividing the difference by the number of individuals who received the first dose of the vaccine (denominator). This proportion was then multiplied by 100 to obtain the percentage dropout [16]. Access to rotavirus vaccination (the children who are reached by immunization services) was measured using percentage coverage and described as either good (≥80%) or poor (<80%). Utilization of rotavirus vaccine (the ability to retain the children accessed by the vaccine until they receive the last dose on the schedule) was measured using the dropout rate and described as either high (when the dropout rate was <10%) or low (when the dropout rate was ≥10%) [16].

**Table 1:** Rotavirus vaccine targets, doses administered, and coverage in Kiambu and Mbita sub-counties, August 2014–April 2016

Sub-county	Year	Monthly target^a^	Yearly target^b^	No. of dose 1^c^	% coverage for dose 1	No. of dose 2^d^	% coverage for dose 2	Dropout (%)
Kiambu	2014	285	1425	1530	107.4	1174	82.4	23.3
	2015	285	3420	4351	127.2	3977	116.3	8.6
	2016	295	1180	1613	136.7	1467	124.3	9.1
	Total		6025	7494	124.4	6618	109.8	11.7
Mbita	2014	402	2010	1096	54.5	667	33.2	39.1
	2015	414	4968	3821	76.9	3348	67.4	12.4
	2016	426	1704	1327	77.9	1156	67.8	12.9
	Total		8682	6244	71.9	5171	59.6	17.1

^a^Number of children aged <1 year being targeted for rotavirus vaccination in a month in each sub-county

^b^Number of children aged <1 year being targeted for rotavirus vaccination in a year in each sub-county. 2014, 2015, and 2016 totals include 5 months (August–December), 12 months (January–December), and 4 months (January–April), respectively, and form the denominator for calculating the vaccine coverage

^c,d^Number of individuals vaccinated with rotavirus doses 1 and 2 in a year in each sub-county. 2014, 2015, and 2016 totals include 5 months (August–December), 12 months (January–December), and 4 months (January–April), respectively, and form the numerator for calculating the vaccine coverage

^a,b,c,d^Compiled from administrative data obtained from the Unit of Vaccines and Immunization Services (UVIS), Kiambu and Mbita sub-counties

### Results

There was a distinct variation in the extent of rotavirus vaccine coverage between Kiambu and Mbita sub-counties. In Kiambu sub-county, the vaccine coverage for 2014 was 107.4% for dose 1 and 82.4% for dose 2, representing a dropout rate of 23.3%. In 2015, the coverage for doses 1 and 2 was 127.2 and 116.3%, respectively, with a dropout rate of 8.6%. In 2016, the coverage was 136.7 and 124.3% for doses 1 and 2, respectively, with a dropout of 9.1% (Table 1). Overall, the coverage for doses 1 and 2 in Kiambu sub-county was 124.4 and 109.8%, respectively, with a dropout rate of 11.7% (Table 1). Thus, the high coverage (≥80%) demonstrates good access to the rotavirus vaccine in this sub-county whereas the high dropout rate (≥10%) indicates a slightly poor utilization of the vaccine in this area.

**Fig. 1:**
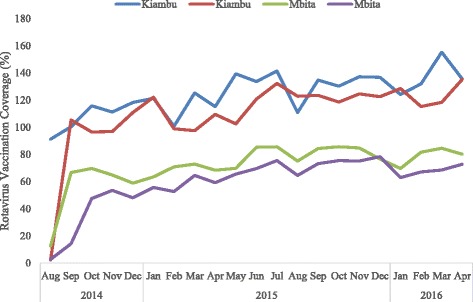
Trends in monthly rotavirus vaccination coverage in Kiambu and Mbita sub-counties, 2014–2016. The graph compares the trends in monthly rotavirus immunization coverage rates between Kiambu and Mbita sub-counties as estimated from the vaccine administrative data for doses 1 and 2 between August 2014 and April 2016. The vaccine administrative data was used with permission from the Kiambu and Mbita sub-counties’ Units of Vaccines and Immunization Services (UVIS)

In Mbita sub-county, rotavirus vaccination coverage for 2014 was 54.5% for dose 1 and 33.2% for dose 2, representing a dropout rate of 39.1%. In 2015, the coverage increased for both doses 1 and 2 at 76.9 and 67.4%, respectively, with a dropout rate of 12.4%. In 2016, the coverage increased to 77.9 and 67.8% for doses 1 and 2, respectively, with a dropout of 12.9% (Table 1). On average, the coverage in Mbita sub-county for doses 1 and 2 was 71.9 and 59.6%, respectively, with a dropout rate of 17.1% (Table 1). Thus, Mbita sub-county recorded a slightly poor access and utilization of the rotavirus vaccine. Both sub-counties recorded a steady increase in monthly rotavirus vaccination coverage for both dose 1 and dose 2 over the study period (Fig. 1).

### Discussion

In this report, we demonstrate varied rotavirus vaccine coverage between Kiambu and Mbita sub-counties in Kenya which are socio-economically different. Kiambu sub-county recorded higher rotavirus vaccination coverage than Mbita sub-county. Disparities in coverage for other childhood vaccines have been reported from various Demographic and Health Surveys (DHSs) in Kenya and have been attributed mainly to the socio-economic status of the child’s family and maternal occupation and education [14, 17–19]. In a recent nationwide DHS, full immunization coverage, especially for the series vaccines, was highest among children from households in the highest wealth quintile (71%) [14]. Kiambu sub-county is wealthier than Mbita sub-county [13–15]. Furthermore, the countrywide survey reported higher immunization coverage in Kiambu than Mbita for both the first and the second doses of the pentavalent, polio, and pneumococcal vaccines [14] that are given alongside the rotavirus vaccine. These factors might explain the higher coverage of rotavirus vaccine in Kiambu than Mbita. The high coverage in Kiambu sub-county indicates good access to the vaccine and might be attributed to enhanced social mobilization within the community. The sub-county UVIS organizes monthly community outreach programs aimed at sensitizing the community on the importance of vaccinations among other health matters, thereby generating demand for vaccines and improving the visibility of immunization among the community. In addition, several community health officers (CHOs) and community health volunteers (CHVs) are engaged in conducting health talks in the community and tracing vaccine defaulters, which have also helped lower the dropout rate.

On the other hand, Mbita sub-county recorded a low rotavirus vaccine coverage, suggesting a poor access to the vaccine. The low coverage might be attributed to the delayed introduction of the rotavirus vaccine, adherence to age restrictions, shortage of health workers, frequent human migrations, and limited cold-chain capacity at some facilities in the sub-county [20]. However, the coverage improved with subsequent years partly due to an enhanced social mobilization and door-to-door health promotion activities by the sub-county’s UVIS. The vaccine dropout was more pronounced for Mbita sub-county, indicating poor utilization of the rotavirus vaccine in this area. Various DHSs in Kenya have observed that the coverage for series vaccinations declined with subsequent doses [14, 17, 19]. The high vaccine dropout in Mbita sub-county might be partly attributed to the constant mobility of the fishing community in the sub-county. Thus, the sub-county CHOs and CHVs should liaise with the fishing community leaders in the public health education programs and in the follow-up of vaccine defaulters.

In this report, vaccine administrative data obtained at the sub-county level were used to estimate the rotavirus vaccine coverage in Kiambu and Mbita. However, there are limitations to the accuracy of immunization coverage estimates derived from the administrative data [21–24]. Hence, the coverage recorded in this report for the rotavirus vaccine could be higher than the actual coverage due to a possible overestimation of the numerators and/or underestimation of the denominators. Overestimation of the numerators might occur due to over-reporting from the peripheral health facilities. In Kenya, the accuracy of the proportion of immunizations reported at the central (sub-county/national) level that can be tracked down to the peripheral health facilities, expressed as the verification factor (VF), has been found to be 85% (95% CI, 68–103%) [23]. We could not verify the coverage data from the peripheral health facilities that were reported to the sub-counties’ UVIS. Overestimation of the numerators could also occur as a result of vaccinating children outside the target age group or target catchment population. Underestimation of the denominators could result from inaccurate vaccine target setting due to population movements and/or inaccurate census projections—Kenya conducts a national census only after 10 years. Thus, the accuracy of the administrative coverage estimates should be improved through regular data quality assessments to avoid errors in recording, compiling, and reporting vaccinations at the peripheral health facilities to higher levels and accurate vaccine target setting by accurate projections of the catchment population. In addition, vaccination coverage surveys should be conducted to verify the administrative coverage estimates [21–24].

## Conclusions

There was a disparity in rotavirus vaccine coverage between the two socio-economically distinct sub-counties; hence, efforts should be made to strengthen the immunization system to facilitate equitable delivery of vaccination services across the country. The high vaccine dropout in both sub-counties underscores the need for enhanced community outreach programs that will encourage those who receive the first dose of the vaccine to return for the remaining dose and facilitate effective tracing of any vaccine defaulters.

## Abbreviations

CHOs: Community health officers
CHVs: Community health volunteers
DALY: Disability-adjusted life year
DHS: Demographic and Health Survey
EPI: Expanded Program on Immunization
GDP: Gross domestic product
KDHS: Kenya Demographic and Health Survey
KEMRI: Kenya Medical Research Institute
UNICEF: United Nations Children’s Fund
USD: United States dollar
UVIS: Unit of Vaccines and Immunization Services
VF: Verification factor
WHO: World Health Organization
